# Comparative feedstock analysis in *Setaria viridis* L. as a model for C_4_ bioenergy grasses and Panicoid crop species

**DOI:** 10.3389/fpls.2013.00181

**Published:** 2013-06-19

**Authors:** Carloalberto Petti, Andrew Shearer, Mizuki Tateno, Matthew Ruwaya, Sue Nokes, Tom Brutnell, Seth DeBolt

**Affiliations:** ^1^Plant Physiology, Department of Horticulture, College of Agriculture, Food and the Environment, University of KentuckyLexington, KY, USA; ^2^Department of Biosystems and Agricultural Engineering, University of KentuckyLexington, KY, USA; ^3^Enterprise Institute for Renewable Fuels, Donald Danforth Plant Science CenterSt. Louis MO, USA

**Keywords:** *Setaria*, cell wall, lignocellulose, Panicoideae, biofuel, cellulose synthase

## Abstract

Second generation feedstocks for bioethanol will likely include a sizable proportion of perennial C4 grasses, principally in the Panicoideae clade. The Panicoideae contain agronomically important annual grasses including *Zea mays* L. (maize), *Sorghum bicolor* (L.) Moench (sorghum), and *Saccharum officinarum* L. (sugar cane) as well as promising second generation perennial feedstocks including *Miscanthus*×*giganteus* and *Panicum virgatum* L. (switchgrass). The underlying complexity of these polyploid grass genomes is a major limitation for their direct manipulation and thus driving a need for rapidly cycling comparative model. *Setaria viridis* (green millet) is a rapid cycling C4 panicoid grass with a relatively small and sequenced diploid genome and abundant seed production. Stable, transient, and protoplast transformation technologies have also been developed for *Setaria viridis* making it a potentially excellent model for other C4 bioenergy grasses. Here, the lignocellulosic feedstock composition, cellulose biosynthesis inhibitor response and saccharification dynamics of *Setaria viridis* are compared with the annual sorghum and maize and the perennial switchgrass bioenergy crops as a baseline study into the applicability for translational research. A genome-wide systematic investigation of the cellulose synthase-A genes was performed identifying eight candidate sequences. Two developmental stages; (a) metabolically active young tissue and (b) metabolically plateaued (mature) material are examined to compare biomass performance metrics.

## INTRODUCTION

As the depth of knowledge ever increasingly grows in the model plant *Arabidopsis* and the utility of this information proves translatable, there remains a need for additional model plants, to decode and translate traits that are absent in *Arabidopsis* (Rensink and Buell, 2004). Monocotyledonous taxa (grasses) display both marked phenotypic and metabolic variability. Moreover, they produce the majority of the world food crops and a growing need for biomass for biofuel production is being driven toward perennial grasses. Thus, a notable need exists for a range of model species to ask a great many questions underscoring agronomically important traits. Existing examples of model grasses include *Brachypodium distachyon* and rice (*Oryza sativa*), which have served as tractable model plants for gene discovery and translational biology (Ouyang et al., 2007; Vogel, 2008). However, there are also important physiological differences among the grasses. A principle difference exists between mechanisms of photosynthesis. Rice and *Brachypodium* utilize C_3_ (carbon fixation) photosynthesis whereas the existing and most promising bioenergy feedstocks including maize (*Zea mays*), sorghum (*Sorghum bicolor*), switchgrass (*Panicum virgatum* L.), or *Miscanthus* (*Miscanthus* × *giganteus*) employ C_4_ (carbon fixation) photosynthesis (Schmitt and Edwards, 1981; Oula, 2008; Wang et al., 2009). While *Setaria* has been proposed as models for C_4_ photosynthesis (Doust et al., 2009; Brutnell et al., 2010; Li and Brutnell, 2011), based on it’s close phylogenetic relationship and genetic synteny, it also exists as an excellent model for the lignocellulosic biomass crops. For perspective, maize has a large genome comparable in size to the human genome, while *Setaria* is one-fifth the size and contains all the genes necessary for C4 photosynthesis and primary metabolic processes. Primary cell walls in dicots and monocots are inherently different. For instance in dicots, cellulose fibers are encased in a matrix of xyloglucan, pectin, and structural proteins. On the contrary, a typical monocot primary cell wall still has cellulose microfibrils as the main structural biopolymer, but has a hemicellulose structure comprised of glucuronoarabinoxylans along with hydroxycinnamates and a very low contribution of pectin (see Vogel, 2008). Differences between the C_3_ and C_4_ plants, in our case focused solely monocotyledonous (monocots) examples (some dicotyledonous C_4_ plants exist), involve structural (physiologically) and metabolic advances in leaf anatomy and atmospheric carbon during photosynthesis. Despite the large evolutionary adaptation in plant physiology between C_3_ and C_4_ metabolism, only minor differences are noted in the cell walls, such as suberized bundle sheath cell walls to prevent CO_2_ leakage (Langdale, 2011). Little evidence exists that C_3_ versus C_4_ metabolism is accompanied by any marked shifts in the composition of cell wall polysaccharides comprising lignocellulosic biomass. However, the primary metabolic response(s) to environmental constraints, such as drought (Doust et al., 2009), are notable different in plants with C_3_ versus C_4_ metabolism and therefore the feedback regulation of carbon through primary metabolism is feasibly influenced. Such shifts could in turn influence cell wall processes. Indeed, in a study by Hibberd and Quick (2002), the spatially discreet evolution of C_4_ metabolism around xylem vessels in a C3 plant was associated with both a pH shift and provision of carbon skeletons into lignin synthesis for rapid incorporation in lignified xylem vessels. Regardless, the development of physiological, compositional, and genetic resources in *Setaria* as a model organism for lignocellulosic feedstocks should complement resources in *Arabidopsis* and *Brachypodium*. Briefly, *Setaria*, is the weedy counterpart to the grain crop *Setaria italica* (millet), more commonly known as the green foxtail. Many crop grasses have vast and repetitive genome structure (Vicient et al., 2001; Bennetzen, 2007). By contrast, *Setaria viridis* has a relatively manageable genome and yet displays synteny to the Panicoideae grasses, which include the major biofuel grasses (Li and Brutnell, 2011). *Setaria* (as referred to from this point onward) displays a life cycle of around 8 weeks, produces abundant seed each generation, is self-compatible and is a diploid providing for genetic tractability (Doust et al., 2009).

**FIGURE 1:**
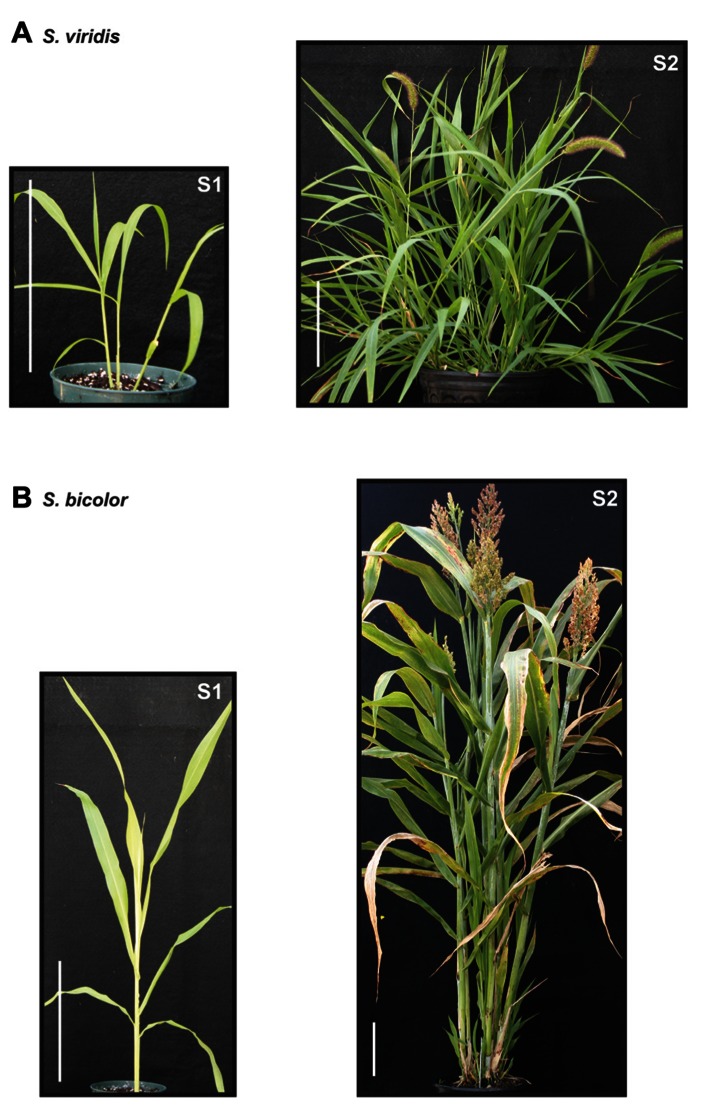
**Representative sample points exemplified during the developmental program of *Setaria* and sorghum.** Plants were sampled at two stages: immature tissue denoted as stage 1 (S1) and mature tissue denoted as stage 2 (S2), exemplified here for *Setaria* **(A)** and sorghum **(B)**.

As an initial step in the development of *Setaria* as a model for bioenergy grasses, the cell wall carbohydrate composition of *Setaria* was compared with other crop species in the Panicoideae at two developmental stages; (a) metabolically active young tissue and (b) metabolically plateaued (mature) material (see **Figure 1** for example). Furthermore, employing current genomic data we phylogenetically examine the cellulose synthase-A (CESA) gene family. Metabolically active tissue was examined because such a sampling time is representative of an early season perennial forage production system [Panicoideae clade species such as switchgrass, eastern gamagrass (*Tripsacum dactyloides*) and *Miscanthus* × *giganteus*] and due to being a temporally favorable timeframe to examine phenotypes in a research setting. Secondly, an important comparison is that of mature tissue where genetic manipulations for persistent biomass traits can be compared and potentially translated.

## RESULTS AND DISCUSSION

### *Setaria* AS A MODEL FOR CELL WALL SYNTHESIS IN THE PANICOIDEAE: THE IDENTIFICATION OF TWO DEVELOPMENTAL STAGES THAT PRESENT SIMILAR CELL WALL COMPOSITION

#### Cellulose, neutral sugars, and lignin

**FIGURE 2:**
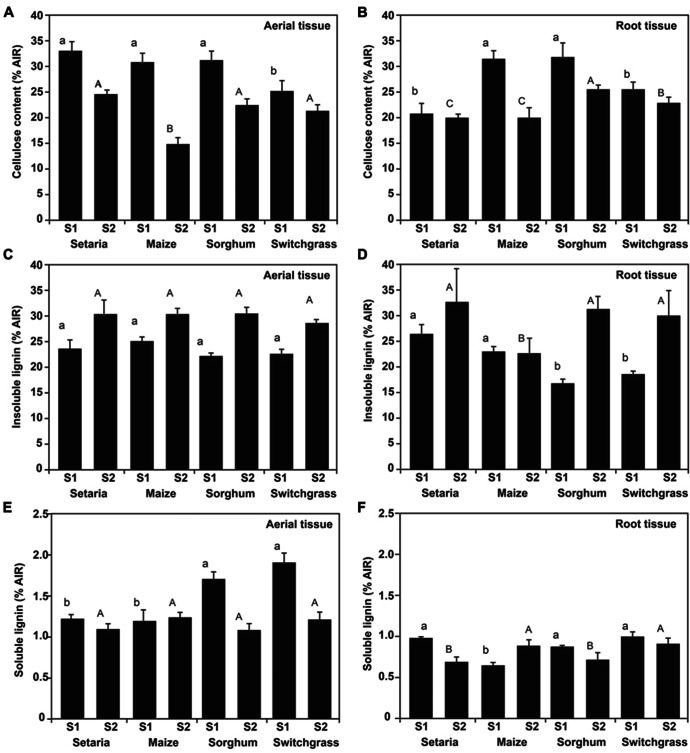
**Cellulose and lignin determination of acid-insoluble residue (AIR).** Cellulose content was determined in aboveground **(A)** and belowground **(B)** material for *Setaria*, maize, sorghum, and switchgrass at both stage S1 and S2. The acid-insoluble lignin portion of aboveground **(C)** and belowground **(D)** and acid-soluble lignin **(E,F)** was quantified. *n* = 3 biological replicates. Error bars represent the standard error from the mean. One-way ANOVA with a *post hoc* Tukey test was performed across the four species per stage. Convention adopted is small letters for stage 1 (S1) and capital letters for stage 2 (S2). Same letters indicate non-significance (*P* > 0.05) different letters indicate significant differences (*P* < 0.05).

It is recognized that the relative abundance of cellulose, lignin, and hemicellulose varies depending on tissue type, age, and environmental/biological condition of the plant tissue (Meinert and Delmer, 1977; Rogers and Campbell, 2004). Cellulose, a natural highly abundant macromolecule made up of glucose 1, 4-β-linked is a principle substrate for bioethanol conversion (Hidayat et al., 2012). Further, hemicellulose is also a convertible carbohydrate substrate to alcohol fuels (Saha, 2003). By contrast, the presence of lignin inhibits enzymatic processes (Li et al., 2010; Kim et al., 2011) and it’s down-regulation through genetic approaches has been shown to improve the saccharification efficiency (Chen and Dixon, 2007; Saathoff et al., 2011; Wang et al., 2012). Therefore, we surveyed cellulose, lignin, and neutral sugar composition in *Setaria* and compared these with other annual and perennial members of the Panicoideae. Cellulose and lignin content of acid-insoluble residue (AIR biomass) was quantified. As defined in the methods and materials, we sought two developmental stages for comparative studies. Analysis of cellulose content in immature biomass revealed no significant change (*P* > 0.05) in relative content among the three annual panicoid species (*Setaria*, maize, and sorghum). By contrast, lower cellulose content was measured in switchgrass samples (**Figures 2A,B** ; *P* > 0.05). There are several plausible explanations for these data. Firstly, switchgrass is a perennial warm season grass whereas the other Panicoideae clade species are annual grasses. The modest variation in cellulose accumulation may reflect the ecological and genetic distances between annual and perennial crops. Alternatively, the capacity to synchronize temporal staging (sampling time) may have been congruent for the annual species, but the perennial switchgrass may display minor developmental variation. In prior analysis of numerous upland and lowland switchgrass cultivars, modest variability in cellulose content was observed (Stork et al., 2009), and therefore species level variation could also exist. Comparison of cellulose content in the mature (S2) compared with immature (S1) biomass revealed an expected and consistent proportional decrease during development (**Figures 2A,B** ; *P* > 0.05). This proportional decrease in cellulose content may be a target for future transcriptional re-wiring efforts that aim to increase cellulose without significant cost to the fitness of the plant. It was also found that the degree to which the cellulose content was reduced in mature tissue relative to the metabolically active tissue was greatest in the two highly domesticated species, maize and sorghum.

The majority of lignin was measured as insoluble lignin. Lignin content increased significantly between the first and second sampling in both aerial (**Figure 2C**) and root tissue (**Figure 2D**). Increased lignification during development was consistent with diversification of the primary cell wall into lignified secondary cell walls in maturing stems, leaves, and roots (Redfearn and Nelson, 2003; Parrish and Fike, 2005; Stork et al., 2009). Soluble lignin although representing a minor proportion of cell wall biomass was measured and found to be less predictable (**Figures 2E,F**). For example, in aerial tissue, soluble content did not significantly change in *Setaria* or maize (*P* > 0.05) and significantly decreased in sorghum and switchgrass between S1 and S2 (*P* < 0.05). Collectively, data highlight a contiguous developmental program during the deposition patterns for cellulose and lignin. These data broadly support the utility of *Setaria* to provide model organism characteristics to above-mentioned crop species, taking note of potential differences when comparing annual and perennial grasses.

Biomass was examined using X-ray diffraction (XRD) to infer a relative crystallinity index (RCI; Segal et al., 1959). The RCI is an empirical measure of the relative proportion of the crystalline versus non-crystalline (amorphous) biomass components, and is not a measure of the crystallinity of cellulose (Andersson et al., 2003). All samples fell in a comparable range of 59–69%, consistent with prior studies (Harris and DeBolt, 2008). Interestingly, there was a general trend of increased RCI from S1 to S2 (**Table 1**). Lignification during secondary cell wall synthesis would increase the amorphous signal at 2-theta (Θ) 18° and subsequently influence the RCI determination. Of further note, a large peak consistent with calcium oxalate was evident in all samples at 2-theta (Θ) 26.6° and was more pronounced in S2 (data not shown).

**Table 1:** Relative crystallinity index (RCI) determined for specified spatial and temporal regions of each species.

Species	Aboveground – RCI (%)*		Belowground – RCI (%)*	
	Stage 1	Stage 2	Stage 1	Stage 2
*Setaria*	57.5 ± 1.2	58.6 ± 2.3	52.0 ± 1.9	60.0 ± 3.2
Sorghum	64.2 ± 3.6	69.2 ± 4.5	54.7 ± 2.0	61.1 ± 2.3
Maize	62.5 ± 2.8	61.2 ± 1.8	54.2 ± 2.0	63.5 ± 3.7
Switchgrass	66.6 ± 3.8	68.1 ± 3.6	52.4 ± 1.2	60.0 ± 2.5

*Values reported are the average of n = 3±SE.

**FIGURE 3:**
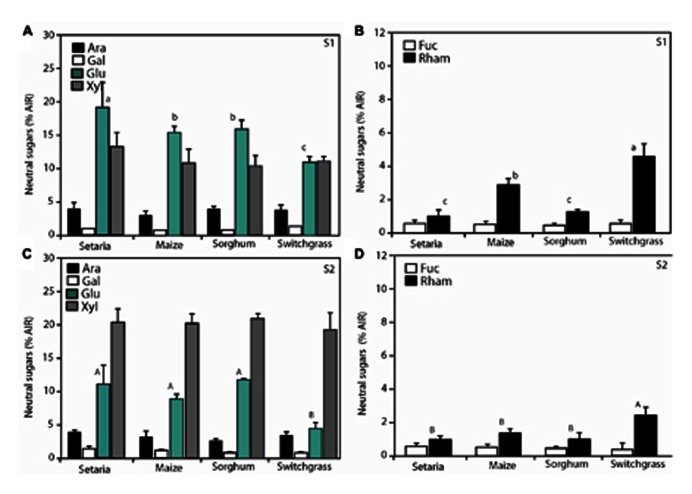
**Neutral sugar monosaccharide analysis for aboveground material.** The relative abundance of each sugar was compared to a true standard and determined *Setaria*, maize, sorghum, and switchgrass at S1 **(A,B)** and S2 **(C,D)**. *n* = 3 biological replicates. Error bars represent the standard error from the mean. One-way ANOVA with a *post hoc* Tukey test was performed across the four species per stage. Convention adopted is small letters for stage 1 (S1) and capital letters for stage 2 (S2). Same letters indicate non-significance (*P* > 0.05) different letters indicate significant differences (*P* < 0.05).

To further understand how the cell wall was changing over time, we measured neutral sugars in S1 (**Figures 3A,B**) and S2 (**Figures 3C,D**). Neutral sugars collectively comprise the building blocks for hemicellulose biosynthesis in the plant cell walls (Bauer et al., 1973) such as xyloglucans, xylans, mannans and glucomannans, and β-(1 → 3,1 → 4)-glucans (Scheller and Ulvskov, 2010). These polysaccharides make up a third class of cell wall biomass component, in addition to cellulose and lignin and comprise arabinose, mannose, rhamnose, galactose, fucose, xylose, and soluble glucose. In S1 aboveground biomass, rhamnose was more abundant in the switchgrass biomass relative to annual species (**Figure 3A**). By contrast, arabinose, galactose, fucose, glucose, and xylose did not display notable shifts in relative abundance (% AIR or mg g^-^^1^). For stage S2, some variability in xylose, glucose, and rhamnose content was observed among genotypes, but this was also most pronounced in switchgrass (**Figure 3**). Mannose and galactose were not abundantly represented in our analysis. These can be explained by (glucurono)arabinoxylans being the predominant component of hemicelluloses in the grasses, while xyloglucans dominate in the cell walls of dicotyledons (including gymnosperms; Scheller and Ulvskov, 2010). The proportional shifts between species herein were minor and no pronounced shifts in total neutral sugars between S1 and S2 were observed. These data support that the increased RCI in S2 (**Table 1**) may be consistent with increased order or abundance of crystalline cellulose within the cell wall rather than a proportional shift in hemicellulose or lignin. The use of hemicellulosic neutral sugars as a feedstock for enzymatic conversion to biofuel requires different degradation enzymes than cellulose (Saha, 2003). The xylose and arabinose sugar backbones represented the most abundant non-glucose sugars available for bioconversion (**Figure 3**). Alternatively, recent innovations show that integrated thermochemical deconstruction of cellulosic and hemicellulosic fractions of lignocellulosic biomass can be simultaneously converted to levulinic acid and furfural, respectively (Alonso et al., 2013), and would not require separate bioprocesses. Ultimately, thermochemical deconstruction of lignocellulosic biomass to biofuel may negate modest compositional shifts, but technical hurdles remain in this maturing technology.

#### Cellulose biosynthesis inhibitor response studies

A chemical inhibitor can be a useful research tool, particularly when combined with genomic tools and model system resources (Robert et al., 2009; Töth and van der Hoorn, 2010). Cellulose biosynthesis inhibitors (CBI) have been effectively used with the dicotyledonous model system *Arabidopsis thaliana* to identify mutations in CESA (Scheible et al., 2001; Desprez et al., 2002), some of which have led to beneficial deconstruction characteristics of cellulose (Harris et al., 2012). CBIs have also been used to learn important information about the delivery mechanisms of cellulose biosynthetic machinery (Crowell et al., 2009; Gutierrez et al., 2009), which will ultimately be important to regulate cellulose synthesis in plants.

**FIGURE 4:**
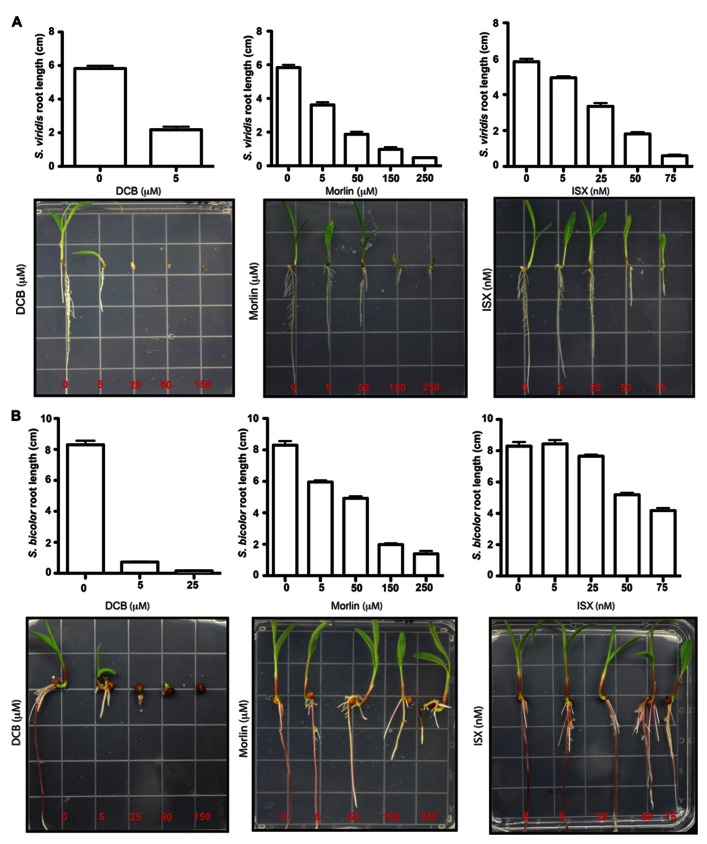
**Comparing response to cellulose biosynthesis inhibitors (CBIs) in *Setaria* and sorghum.** Response to 2,6-dichlorobenzonitrile (DCB), morlin (7-ethoxy-4-methyl chromen-2-one), and isoxaben (*N*-[3-(1-ethyl-1-methylpropyl)-1,2-oxazol-5-yl]-2, 6-dimethoxybenzamide) from switchgrass **(A)** and sorghum **(B)** was examined during early seedling growth using in plate assays on sterile Murashige and Skoog (MS)-agar media. Average root length was measured (*n* ≥ 10) and error bars represent standard error from the mean.

In plants, directional cell growth is facilitated by a rigid, yet extensible cell wall, which acts to collectively constrain internal turgor pressure. Cellulose forms the central load-bearing component of this wall and therefore inhibiting cellulose biosynthesis causes radially swollen cells in seedlings providing a robust visual phenotype drug activity. A panel of CBIs was tested for potency herein, due to their possible use in future studies on biomass traits. These CBIs fell into three different classes of inhibition mechanism in *Arabidopsis* (summarized in Brabham and DeBolt, 2013), clearance of CESA (isoxaben (*N*-[3-(1-ethyl-1-methylpropyl)-1,2-oxazol-5-yl]-2,6-dimethoxybenzamide)), stopping CESA (2,6-dichlorobenzonitrile – DCB) and co-targeting cortical microtubules and CESA [morlin (7-ethoxy-4-methyl, coumarin); **Figure 4**]. The LD_50_ dose rates for isoxaben, morlin, and DCB in *Arabidopsis* are 4 nM, 500 nM, and 5 μM (Heim et al., 1989; DeBolt et al., 2007a,b). Results showed that isoxaben failed to induce radial LD_50_ root length suppression until 50–100 nM (**Figure 4**), which represented a 15- to 20-fold reduction in activity. Morlin also displayed a 10-fold reduction in activity, with an LD_50_ of approximately 50 μM. Based on these data, DCB alone remained a highly potent CBI inhibitor for the panicoid with dose rate similar to those observed in *Arabidopsis*. The lower degree of CBI potency observed for isoxaben and morlin, but not for DCB, requires future exploration. Indeed, ^14^C-isoxaben studies from Heim et al. (1993) suggest that isoxaben uptake is not blocked at the cuticle in monocotyledons. Further, the action of DCB does not support a non-specific biochemical drug tolerance such as more abundant cytochrome P450 enzymes (Morant et al., 2003). Hence, future chemical genetic studies employ mutagenesis populations to map based clone resistance loci to CBIs are needed. Such studies will be aided by a genetic model such as *Setaria*.

#### Phylogenetic analysis of CESA genes

**FIGURE 5:**
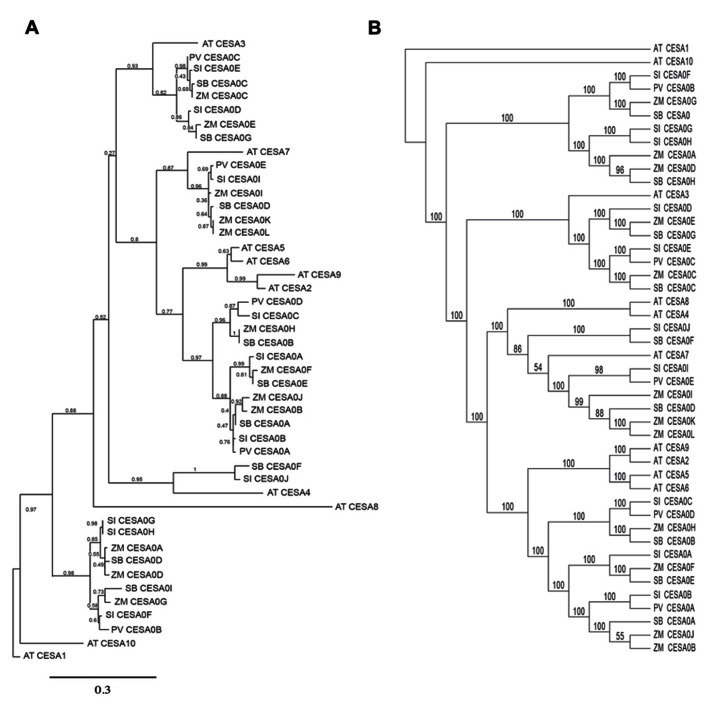
**Phylogenetic trees of CESA proteins (A)** and *CESA* genes **(B)**. Phylogenetic relationships were determined for *A. thaliana* (AT), *Setaria italica* (SI), *Z. mays* (ZM), *Sorghum bicolor* (SB), and *P. vulgare* (PV).

In order to ask whether similarities in cellulose biosynthesis were reflected by similar organization of the CESA gene family, we performed a simple phylogenetic analysis of annotated genes. To aid in providing confidence in the bioinformatic analysis, two separate phylogenetic trees were established using distinct protein prediction compared with genomic sequence. Homology-based associations were made between *CESA* identified in the genomic data derived from *A. thaliana* (AT), *Setaria italica* (SI), *Z. mays* (ZM), *Sorghum bicolor* (SB), and *P. vulgare* (PV). Firstly, the MUSCLE multiple alignment tool (Edgar, 2004) was utilized followed by the PhyML program (Guindon et al., 2010) to create the phylogenetic tree based on the translational predictions (protein sequences) of the putative CESAs. GARLI was utilized in parallel to produce a phylogenetic tree based on genomic sequence information. When grouping *CESA* genes or gene products within the broad CESA phylogenetic tree, it was necessary to visualize groups (sub-clades) of putative CESAs within the tree, and therefore they were referred to based on the closest homology to the established *Arabidopsis* CESA nomenclature of Richmond (2000). The phylogenetic tree inferred by protein sequences (**Figure 5**) clustered in five classes with the *Arabidopsis* CESA1, 3, 7, 6-like, and 4. It was perhaps noteworthy that the *Arabidopsis* CESA6-like clade, which also comprised proteins similar to *Arabidopsis* CESA2, 5, and 9 (Persson et al., 2007) was the most highly represented cluster. By contrast, the Panicoideae homologs with *Arabidopsis* secondary cell wall CESA4 were least common, which only *Z. mays* and *Setaria* containing putative members of the CESA4-associated clade. With the exception of the *Arabidopsis* CESA4 clade-comprising of only two putative CESA members, the remaining clades contain at least one putative CESA member from each of the five species investigated. Using genomic data to assemble and predict phylogenetic relationships (**Figure 5**), the putative CESAs divide readily into four distinct clades, clustering around *Arabidopsis* *CESA1* (primary cell wall; Arioli et al., 1998), *CESA3* (primary cell wall; Scheible et al., 2001), *CESA4/7/8* (vascular secondary cell wall; Taylor et al., 2003), and *CESA6-like* (*CESA2*, *5*, and *9*, included in primary and seed coat secondary cell wall; Stork et al., 2010; Mendu et al., 2011; Sullivan et al., 2011). It was found that at least one putative CESA member from each of the five species was represented within each sub-clade. However, in the heterotrimeric model for cellulose synthase complex (CSC) formation in the primary and secondary cell wall (Delmer and Amor, 1995; Tanaka et al., 2003), the lack of at least three distinct CESAs within the *CESA4*, *7*, and *8* sub-clade suggests either divergence among heterotrimeric associations or an example of large incompletions within the genome sequencing for all of the Panicoideae. In comparing the two phylogenetic trees, there were no marked differences in the CESAs that comprised a particular sub-clade. For example, in both phylogenetic trees the *Arabidopsis* *CESA6*-like *CESA2*, *5*, *6*, and *9* cluster closely with each other and were broadly represented. The discrepancy in the number of clades between the two trees can be attributed to the fact that within the protein sequence phylogenetic tree, the *Arabidopsis* CESA4 clade and CESA7 clade are separated while they are combined into one clade within the genetic sequence phylogenetic tree. Ultimately, targeted reverse genetics are urgently needed in the model grasses to elucidate questions related to the tissue specificity, functionality, redundancy, and stoichiometry of the CESA genes. Further, next generation sequencing to produce in depth transcriptional patterns for spatially and temporally diverse tissues could reveal whether differentiation during caryopsis development utilizes *CESA6-like* sequences in a non-redundant manner as observed in *Arabidopsis* (Mendu et al., 2011; Sullivan et al., 2011).

### DEGRADABILITY

**FIGURE 6:**
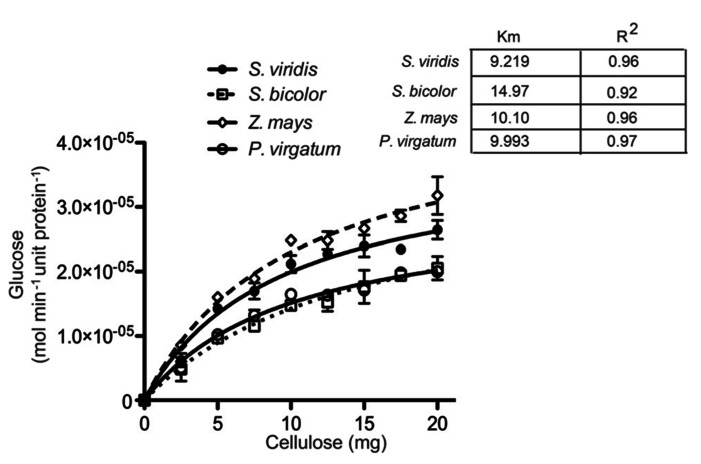
**Pseudo-apparent kinetics and cellulosome biodegradation of total biomasses.** Michaelis–Menten enzymatic kinetics were determined for semi-purified cellulose for the aboveground stage 2 (S2) of *Setaria viridis* (SV); *Z. mays* (ZM); *Sorghum bicolor* (SB), and *P. vulgare* (PV).

Saccharification efficiency was measured for the four species as a criterion for utilizing lignocellulose biomass to produce alcohol-based biofuels. Several different tests were performed to determine the dynamics and cross species consistency of digestibility. Firstly, we examined the pseudo-apparent kinetics of digestibility in semi-purified cellulose (Harris et al., 2009) from the mature stem (S2) of each panicoid (**Figure 6**). Results showed a consistent rate of conversion of cellulose to fermentable sugars, with sorghum displaying a notable increase in digestibility.

**FIGURE 7:**
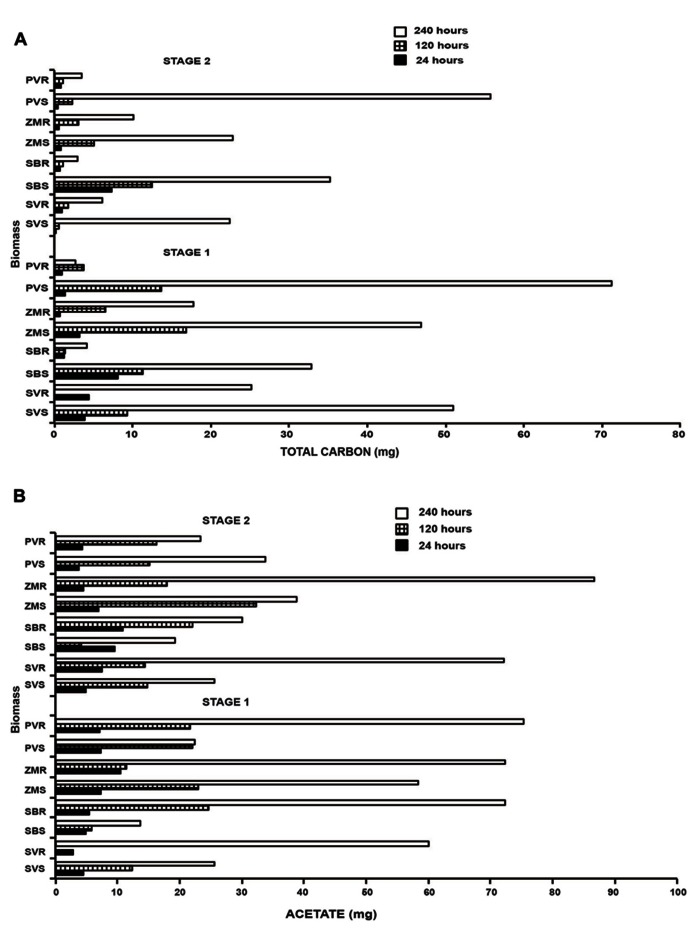
**Total carbon (A) and lactate (B) production obtained through *Clostridium thermocellum* system during biomass deconstruction.** In **(A)**, biological degradation of the four grasses was completed through *Clostridium thermocellum* system. The saccharification efficiency was determined for stage 1 and 2 (S1, S2) and for aboveground and belowground material for *Setaria viridis* (SV); *Z. mays* (ZM); *Sorghum bicolor* (SB), and *P. vulgare* (PV). In **(B)**, The graph displays the acetate production for stage 1 and 2 and for three time points (24, 120, and 240 h) representing the initial stage the middle and the final stage of the process. The convention employed in the graph was: name of species plus S (S denotes shoot) for the aboveground and R (R denotes root) for the belowground material.

We subsequently used a cellulosome digestion via *Clostridium thermocellum* and measured the output of carbon from raw unprocessed biomass. During the saccharification reaction two main products are generated, glucose and cellobiose. Additionally, *C. thermocellum* is capable of metabolizing glucose and cellobiose, and acetate, lactate and ethanol are products of this metabolism. It is possible to estimate the total amount of carbon released from the biomass as the result of the simultaneous saccharification and fermentation process by the inclusion of all the products, thus accounting for the total carbon signature of the converted biomass. Time course fermentation (from 0 to 240 h) released an increased amount of carbon as time progressed (**Figure 7A**). For both stages, we observed a similar increase in the temporal series; although, as expected for stage 2, a lesser amount of total carbon was released (**Figure 7A**). Three noteworthy trends were observed; (1) *Setaria* displayed the second highest rate in stage 1 and the highest total carbon conversion for the root material. (2) The highest rates of total carbon release was, for both stages, observed in switchgrass, and (3) the values determined for maize and sorghum were intermediate. The most interesting aspect, when single fermentation products were taken into account was related to the production of acetate whereby the highest release was associated with the fermentation of the root biomass independently of the species considered although, it was most obvious at the 240 h time point than the other time assayed (**Figure 7B**). This was the only case where the root biomass conversion products were higher than the conversion products from the shoot biomass.

## CONCLUSION

Several studies highlight *Setaria* as a model organism for the panicoid (see Li and Brutnell, 2011), complementing the existing resources for C3 grasses via rice and *Brachypodium*. In this study, we examined the baseline composition, CBI response, and CESA gene family in *Setaria.* Data indicated that commonly used CBIs for dicot studies, such as isoxaben as less effective for *Setaria*, whereas DCB displays similar LD50 responses. Eight members of the CESA gene family were identified for functional genomic characterization. The use of two growth stages demonstrated the potential translational role of *Setaria* as a model species for lignocellulosic panicoid crops. To summarize, aboveground *Setaria* biomass was similar to the comparative (non-model) species.

## MATERIALS AND METHODS

### PLANT GROWTH CONDITION AND MATERIAL COLLECTION

Four grasses species were considered and include maize-hybrid 684GENSS, sorghum var. Della, *Panicum virgatum* switchgrass var. Alamo and *Setaria*. Seeds were sown on soil less media (MetroMix 360, SunGro Industries Bellevue, WA, USA). Plants were grown and maintained in a temperature-controlled glasshouse at 24°C, on a 16 h:8 h light:dark cycle with additional supplemental light if natural light was absent. MetroMix 360 media was maintained at field water conditions and fertilized with 3 g of osmocote (The Scotts Company, Marysville, OH, USA) integrated into the media when planting. A physiological staging was found to be most appropriate, as opposed to temporal staging due to inherent variation in reproductive cycle. Stage 1 was defined by four fully developed leaves (not including the cotyledon) and evidence of culm elongation and stage 2 (mature) determined by the onset of apical inflorescence protrusion. Plant specimen were divided into aboveground and belowground material. Each sub-sample was air dried for two days at 26°C then oven dried at 45°C for a further 7 days. Approximately 10 samples were pooled for each replicate. Four biological replicates of randomly harvested material were sampled per species and per developmental stage. Each biological replicate was made of pooled samples per species and per stage. After oven drying, shoot and root material was ground using to 1 mm fragments (Arthur H. Thomas Co Scientific, Philadelphia, PA, USA) and used for further analytical determinations detailed below.

### CELL WALL CARBOHYDRATE COMPOSITIONAL STUDIES

Cellulose content for all samples was measured colorimetrically (Updegraff, 1969). Neutral and acidic sugar composition was determined by high performance liquid chromatography (HPLC; Mendu et al., 2011). In brief, samples were homogenized using a grinder (Arthur H. Thomas Co Scientific, Philadelphia, PA, USA) equipped with a 1-mm sieve. Twenty-five milligrams plant material were destarched as by the specification in National Renewable Energy Laboratory (NREL), LAP-004 (1996) guidelines by incubating the samples in 1 ml 70% ethanol overnight at 65°C, washed twice with 1 ml 70% ethanol for 1 h and once with 1 ml acetone for 5 min. The samples were dried overnight at 30°C with shakings. Cellulose content was determined by weighing out 5 mg of dry biomass extract in quadruplicate and boiled in acetic-nitric reagent (acetic acid:nitric acid:water 8:1:2) for 30 min to remove lignin and hemicellulosic carbohydrates (Updegraff, 1969). Remaining material was then washed twice with 8 ml MQ-water and 4 ml acetone and dried under vacuum. The cellulose samples were then hydrolyzed in 67% sulfuric acid for 1 h. The glucose content of the samples was determined by the anthrone method (Updegraff, 1969). Ten microliters of the sulfuric acid hydrolyzed samples were mixed with 490 μl water and 1 ml 0.3% anthrone in concentrated sulfuric acid on ice. The samples were boiled for 5 min then placed immediately back on ice. The absorbance of the samples was measured using a Bio-Mate Thermo Scientific spectrophotometer (Thermo Fischer, Waltham, MA, USA) set at OD 620 nm and the cellulose content was calculated by multiplying the measured glucose concentration of each sample by the total volume of the assay and then by the hydration correction factor of 0.9 to correct for the water molecule added during hydrolysis of the cellulose polymer.

For monosaccharide analysis, 4 mg of ethanol–acetone washed plant biomass was measured into glass tubes, a total of 36.5 μl of 72% (w/v) H_2_SO_4_ was added including the internal standard Ribose. The samples were incubated on ice for 2 h, mixing every 30 min with the vortex. Samples were diluted to 4% (w/v) H_2_SO_4_ by adding 963.5 μl of water and autoclaved at 121°C for 1 h. Neutral sugar standards (Fuc, Ara, Rha, Gal, Glc, Man, and Xyl) and acid sugar standards (GalUA) were also processed in the same way at the same time. Neutral and acidic cell wall sugars were identified and quantified by pulsed electrochemical detection using a Dionex ED50 (Dionex, Thermoscientific). Sugar separation was achieved using a BioLC GS50 HPLC device and a CarboPAC-PAl pellicular anion-exchange column with column guard (Dionex). Column and detector temperature was maintained at 30°C using a Dionex LC25 chromatography oven. Samples were introduced to the column from a Dionex AS50 autosampler and neutral sugars were eluted isocratically in 22 mM NaOH over a 30-min period, after which the column was washed in 1 M NaOAc and 200 mM NaOH for 5 min. The column was recharged by passing 200 mM NaOH through it for 10 min and then reequilibrating the column in 22 mM NaOH for 10 min prior to injection of the next sample. This protocol resulted in optimal (near-baseline) resolution of Gal, Glc, Man, and Xyl (Downie and Bewley, 1996). Acidic sugar separation and quantification used the same HPLC procedure but followed the eluent profile of Dean et al. (2007). For total biomass monosaccharides, the neutral sugars were normalized to the amount of plant material (aboveground and belowground) used for the sample preparation.

### LIGNIN QUANTIFICATION

#### Acid hydrolysis

Acid-soluble lignin, acid-insoluble lignin, and ash were measured using the laboratory analytical protocols NREL, LAP-004 (1996). Briefly, approximately 300 mg sample^-^^1^ (±0.01 g) of dry plant tissue were placed into 30 ml test tubes with three replicates followed by an addition of 3 ml of sulfuric acid (72% v/v) to each test tube and stirred thoroughly with a glass rod. Samples were incubated in a water bath at 30°C for 2 h and stirred every 15 min. After hydrolysis, samples were diluted with 84 ml of deionized water (4% concentration) into a 250-ml Erlenmeyer flask that was autoclaved at 121 °C at 15 psi for 1 h.

#### Acid-soluble lignin measurement

Acid-soluble lignin was measured using NREL, LAP-004 (1996). Briefly, the autoclaved solution was allowed to cool to room temperature before withdrawing 1 ml of the hydrolyzate without disturbing any solid particles in the solution. A 4% solution of sulfuric acid was used as a reference blank. To accommodate the absorbance range (0.2–0.7) of the spectrophotometer at 240 nm, both the hydrolyzate and reference 4% sulfuric acid solution were diluted 1:30 with deionized water. The absorbance of the hydrolyzate at 240 nm using a 1-cm light path cuvette was used to calculate the acid-soluble lignin.

#### Acid-insoluble lignin measurement

Porcelain filter crucibles containing a glass microfiber filter (Whatman 934-AH) were placed in a furnace at 575 °C for 4 h. The crucibles were removed from the furnace and placed into a desiccator to cool for a minimum of 1 h before being weighed. The autoclaved samples were vacuum filtered through the crucibles. The crucibles and their contents were dried in an oven at 105 °C overnight before transferring into a desiccator to cool. The weight of the crucibles were recorded and placed in a furnace at 575°C for 4 h before reducing the temperature to 105°C overnight. The crucibles were removed and placed into a desiccator to cool and weighed.

#### Enzyme kinetic of semi-purified cellulose

Crude cell walls were prepared from total biomass of each species (stage 2) as by published protocol (Reiter et al., 1993) and it was scaled up in order to meet the requirements for enzyme kinetics. Cellulose which was purified according to the method by Updegraff (1969) was homogenized using an Arthur H. Thomas Co Scientific grinder (Philadelphia, PA, USA) equipped with a 0.5-mm sieve. A cocktail of cellulase and cellobiose (Sigma, USA), equivalent to 2 FPU (filter paper unit), were used to digest an increased amount of cellulose substrates (2, 5, 7.5, 10, 12.5, 15, 17.5, and 20 mg) in a 50-mM citrate buffer (pH 4.8) at 50°C for 2 h. Prior to glucose determination, the enzymes were heat-inactivated by boiling the samples for 5 min at 100°C. Glucose quantification was done by using a Yellow Springs Instruments (YSI)-glucose analyzer. The Inability to exactly calculate the number of catalytic ends in the complex mixture of cell wall biomass allowed only for the calculation of relative estimation, expressed as relative kinetics (relative *V*_max_ and *K*_m_ ). The glucose values were converted in mol min^-^^1^ unit protein^-^^1^ and used to determine the apparent kinetics values using the program GRAPHPAD PRISM-4 (Graphpad, La Jolla, CA, USA).

#### Cellulose biosynthesis inhibitor studies

The effect of three CBI, isoxaben (*N*-[3-(1-ethyl-1-methylpropyl)-1,2-oxazol-5-yl]-2,6-dimethoxybenzamide; Dow Chemical Company, Midland, MI, USA), DCB (Sigma Aldrich, St Louis, MO, USA), and morlin (7-ethoxy-4-methyl, coumarin Sigma Aldrich, St Louis, MO, USA) were employed to evaluate the plant responses to increased concentration on an in plate assay. Due to the large-sized maize seeds and the poor germination rate of switchgrass seeds, the assay was carried only for *Setaria viridis* and *Sorghum bicolor*. Seeds were surface sterilized in 1% bleach and 0.1% sodium dodecyl sulfate (SDS), for 15 min, twice with a water wash in between. An additional 70% ethanol wash was completed for 5 min; the seeds were then rinsed 10 times with sterile water. For *Sorghum Bicolor*, an average of 15 seeds were plated onto Hoagland’s based media containing either isoxaben (concentration range: 5, 25, 50, and 75 nM), DCB (concentration range: 5, 25, 50, and 150 μM) and morlin (concentration range: 5, 50, 150, and 250 μM) as well as a mock control plate (dimethyl sulfoxide, DMSO). A similar protocol was employed for *Setaria viridis*, but the number of seeds increased to between 30 and 40. Plates were incubated in a climate-controlled chamber (Conviron, Winnipeg, Canada) at 22°C, with a 16 h:8 h light:dark regimen. Each drug concentration was replicated (*n* = 3) and the effects of the drugs were evaluated on the root length and determined 8 days post-germination.

### ANAEROBIC FERMENTATION ANALYSIS

#### Inoculum preparation and chemicals

*Clostridium thermocellum* ATCC 27405 was provided by Michael Flythe (USDA, Lexington, KY, USA) and was maintained in our laboratory, as described by Johnson et al. (1981). Chemically defined thermophile medium was prepared as described by Dhamagadda et al. (2010). A bacterial culture was grown for 24 h in Balch tubes (pH 6.7) at 63°C containing cellulose strips (Whatman Grade 1 filter paper) suspended in 8.5 ml basal medium. This initial culture was used to inoculate 54 ml of cellulose fiber in 100-ml sealed serum vials (Bellco Biotechnology, Vineland, NJ, USA) under CO_2_ atmosphere. After 18 h of growth, the secondary culture was diluted with fresh basal media (substrate free) to prepare the standard inoculum stock (final optical density of 0.162 OD 600; ~0.078 g dry cells l^-^^1^).

#### Biomass microbial cultures and sampling

One milliliter of prepared inoculum culture was inserted by syringe into a test tube containing 8.5 ml of thermophile medium and 0.5 g of plant material to achieve a 5% w/w bacterial suspension. Test tubes containing plant material and media were tightly sealed with CO_2_ headspace and autoclaved at 120°C for 20 min prior to inoculation. Cultures were incubated at 63°C in a water bath. The control consisted of substrate (0.5 g Avicel), media, and bacteria. One milliliter supernatant (triplicate samples without replacement) was collected after 0, 24, 72, 120, 168, and 240 h of fermentation. Each sample collected was centrifuged (5000 × *g*, 20 min, 4°C) and aliquots of cell free supernatant were stored at -20°C until analysis by HPLC. The samples were HPLC analyzed for the presence of the five main products known to be produced by the bacterium. These were matched to the retention time of true standards. Products were: cellobiose, glucose, acetate, ethanol, and lactate.

#### X-ray diffraction analysis

Finely ground biomass samples were then contained in a custom-built sample holder of pressed boric acid. In brief, plant material was placed into a mold, containing a sleeve and hand pressed with a solid metal plug forming a disk shape. The sleeve and plug were removed and a boric acid (Fischer, Madison, WI, USA) base was then formed by pouring the boric acid over the bottom and sides of the sample and applying 40,000 psi of pressure to the 40 mm × 40 mm mold using a Carver Autopellet Press (Wabash, IN, USA). Samples were pressed to create an even horizontal surface. A Bruker-AXS Discover D8 Diffractometer (Bruker-AXS USA, Madison, WI, USA) was used for wide angle XRD with Cu Ka radiation generated at 30 mA and 40 kV. The experiments were carried out using Bragg-Brentano geometries (symmetrical reflection). Diffractograms were collected between 5° and 35° (for samples with little baseline drift), with 0.02° resolution and 2-s exposure time interval for each step. Sample rotation to redirect the X-ray beam diffraction site was achieved per replicate. The data analysis was carried out using the calculation for RCI of: RCI = *I*_002_ - *I*_am_ /*I*_002_ × 100, where *I*_002_ is the maximum peak height above baseline at approximately 22.5° and *I*_am_ is the minimum peak height above the baseline at ~18°. For assessment of experimental accuracy, the pressed samples were examined using reflective geometries at 22.5° 2-theta with the sample scanned rotationally (360°) and in an arc (90°) to obtain an intensity/spatial orientation plot of a sample for which the RCI had already been established. The range of reflective intensities was then used to estimate the accuracy of the RCI determination using a 95% cutoff across the plot range. Diffractograms were collected in Diffrac-Plus-XRD Commander software (Bruker-AXS, Karlsruhe, Germany) and minimally processed (baseline identification, noise correction, 3D display, and cropping of RCI signature region) using the EVA and TexEval (Bruker-AXS Karlsruhe, Germany) software.

### PHYLOGENETIC ANALYSIS

The sequences of the *A. thaliana* cellulose synthase genes were obtained from the TAIR database^1^ and were utilized as the initial reference in order to obtain the putative CESA genes of the grasses. The cDNA sequences of the ten cellulose synthase genes of *A. thaliana* were employed to conduct the BLASTX (Altschul et al., 1997) search within the *Setaria italica*, *Sorghum bicolor*, *Panicum virgatum*, and *Z. mays* genomes. BLASTX searches were conducted using the phytozome database provided by JGI^2^. Due to the very few number of putative CESA genes for *Panicum virgatum* that resulted from the BLASTX search, an additional BLASTP search was conducted using the 12 CESA genes of the *Z. mays* to determine if there were any additional putative genes. After the identification of the putative CESA genes, the protein sequences of each of the putative genes were screened to determine if they contained the CxxC motif (Richmond, 2000) considered to be unique to the CESA genes and distinguishing them from the cellulose synthase like genes. A total of 36 putative cellulose synthase genes were identified. The putative CESA genes that contained the CxxC motif of the four monocot species as well as the known CESA genes of *A. thaliana* were then uploaded onto the PhyML program (Guindon et al., 2010). The putative CESA gene protein sequences that contained the CxxC motif of the four monocot species above as well as the CESA protein sequences of *A. thaliana* were analyzed using PhyML (v.3.0; Guindon et al., 2010). The advanced setting within PhyML conducts a sequence alignment using MUSCLE (v.3.7; Edgar, 2004), then automatically utilizes the alignment to create the phylogenetic tree. A total of 500 bootstrap replicates were conducted under the WAG (Whelan And Goldman) protein substitution model (Whelan and Goldman, 2001).

In addition to the amino acid-based tree, a tree was constructed using the DNA sequences of the 36 putative CESA genes as well as the 10 *Arabidopsis* CESA genes. The sequences were obtained from the Phytozome database and the TAIR database, respectively. The sequences were submitted to the Guidance web server (Penn et al., 2010) and aligned with MAFFT (Katoh et al., 2005). Guidance is a program that both aligns sequences and assesses confidence in each position in the alignment, in order to objectively identify and remove regions of ambiguous alignment prior to phylogenetic analysis. Any column in the alignment that had a confidence score of less than 95% was removed by Guidance. GARLI (v. 2.0; Zwickl, 2006) was used to analyze the resulting alignment under the GTR + I + G model (Rodriguez et al., 1990), in which a best tree search (five replicates) and a 200-replicate bootstrap analysis were conducted, both using the default settings. The resulting maximum likelihood tree was then visualized via FIGTREE^3^.

### STATISTICAL ANALYSIS

Statistical analysis consisting of one-way analysis of variance (ANOVA) with a *post hoc* Tukey test was performed by using PRISM4 (Graphpad, La Jolla, CA, USA). The outcomes are indicated in letters on the corresponding graphs. Same letter signify non-significance (*P*>0.05) whereas different letters indicate significance (*P*<0.05).

## Confilct of interest statement

The authors declare that the research was conducted in the absence of any commercial or financial relationships that could be construed as a potential conflict of interest.

## Footnotes

1 http://www.arabidopsis.org

2 http://phytozome.net

3 http://tree.bio.ed.ac.uk/software/figtree/
